# Controlled Synthesis of Tellurium Nanowires

**DOI:** 10.3390/nano12234137

**Published:** 2022-11-23

**Authors:** Vladimir Miranda La Hera, Xiuyu Wu, Josué Mena, Hamid Reza Barzegar, Anumol Ashok, Sergey Koroidov, Thomas Wågberg, Eduardo Gracia-Espino

**Affiliations:** 1 Department of Physics, Umeå University, 901 87 Umeå, Sweden; 2 Department of Materials and Environmental Chemistry, Stockholm University, 106 91 Stockholm, Sweden; 3 Department of Physics, Stockholm University, 106 91 Stockholm, Sweden

**Keywords:** tellurium, bismuth, doping, nanowires, physical vapour deposition

## Abstract

One-dimensional tellurium nanostructures can exhibit distinct electronic properties from those seen in bulk Te. The electronic properties of nanostructured Te are highly dependent on their morphology, and thus controlled synthesis processes are required. Here, highly crystalline tellurium nanowires were produced via physical vapour deposition. We used growth temperature, heating rate, flow of the carrier gas, and growth time to control the degree of supersaturation in the region where Te nanostructures are grown. The latter leads to a control in the nucleation and morphology of Te nanostructures. We observed that Te nanowires grow via the vapour–solid mechanism where a Te particle acts as a seed. Transmission electron microscopy (TEM) and electron diffraction studies revealed that Te nanowires have a trigonal crystal structure and grow along the (0001) direction. Their diameter can be tuned from 26 to 200 nm with lengths from 8.5 to 22 μm, where the highest aspect ratio of 327 was obtained for wires measuring 26 nm in diameter and 8.5 μm in length. We investigated the use of bismuth as an additive to reduce the formation of tellurium oxides, and we discuss the effect of other growth parameters.

## 1. Introduction

The use of semiconducting nanowires in fields such as gas sensors, solar cells, electronic devices, and opto-electronic devices [1,2,3,4] has gained significant interest in recent years. Nanostructured tellurium, selenium, and their alloys have shown unique electrical and optical properties [5,6,7,8]. Trigonal Te is a semiconductor material that possesses a narrow and nearly direct band gap (i.e., the difference between the direct and indirect bandgap is smaller than the thermal energy at room temperature) of 0.35 eV [9], and interestingly it has a highly anisotropic growth tendency due to the existence of helical chains of covalently bond atoms [10,11]. However, the most common nanowires used in the semiconductor technology are Si, Ge, or GaAs based. These are usually produced via vapour–liquid–solid at various temperatures, such as 350–1000 °C for Si [12,13], 300–500 °C for Ge [14], and 420–680 °C for GaAs [15,16]. Meanwhile, Te NWs can be easily produced at relatively low temperatures below 250 °C. The ability to grow Te at such low temperature is highly advantageous due to a better compatibility with Si circuit fabrication methods, where 450 °C represents an upper limit for growing structures on top of prefabricated circuits and metal interconnected layers. For integration in organic electronics, an even stricter temperature limit arises from the glass transition temperature of the common polymers used, which is usually around 200 °C. It is further noteworthy that the low band gap of bulk Te is not optimal for certain semiconducting applications, such as transistors. However, nanostructured Te can crystallize in the trigonal phase, and such trigonal Te exhibits high hole mobility (707 cm V^−1^ s^−1^) [17], high current density (1 A mm^−2^) [18], and has tunable energy bandgaps up to 1 eV [19], opening up additional research fields. Several studies have already shown the possibility of obtaining a variety of nanostructures, such as needle-like nanowires with a tip of 50–70 nm and a base of 150–200 nm [20], Te nanorods 300–500 nm in diameter and 3 μm in length [21], nanowires with a base of 200 nm and a conical tip with diameter of 20 nm [22], tubular nanowires with an inner size of 50 nm and an external size of 500 nm [23], and finally hexagonal nanowires with tunable dimensions with diameters of 50–3000 nm and lengths of 1–22 μm [24]. The most common production techniques are based on wet-chemistry processes, while few reports exist on other methods such as chemical vapour deposition (CVD), PVD, or sputtering techniques [11,20].

PVD has been shown to be suitable for tuning the morphology of Te micro- and nano-structures depending on the growth parameters (i.e., the source and deposition temperatures, pressure, and carrier gas flow rate) [20,21]. The guiding principle for vapour phase growth is to control the supersaturation of the vapours by properly tailoring the growing parameters such as the precursors, deposition temperatures, pressure, and carrier gas flow rate. It has been previously reported by Sen, S. et al. [20] and Hyung, J.H. et al. [21] that the morphology of Te nanostructures is highly dependent on the growth temperature and gas flow rate. Even though the obtained nanostructures in both works have large diameters (≥200 nm), they provide an initial approach to understanding the control of the morphology, which is still far from ideal.

Therefore, in this report we aim to synthesise crystalline trigonal Te nanowires by PVD at low temperatures, preferably below 200 °C, and concurrently tune the conditions to achieve nanowires with high aspect ratios. We have established that the nanowires grow along the (0001) direction and that their diameter and length can be controlled so that Te nanowires with an aspect ratio >300 can grow in a relatively precise “growth window” by varying the synthesis conditions. We further show that Te nanowires are prone to oxidation, forming TeO_2_ at the surface, but that this oxidation can be at least partly mitigated by bismuth doping.

## 2. Experimental Details

*Chemicals:* Tellurium powder (200 mesh, 99.999% purity, Fisher Scientific, Kandel, Germany). Bismuth powder (100 mesh, 99.99% purity, Merck KGaA, Darmstadt, Germany). Arcal 15 gas (95% argon and 5% hydrogen), and Argon (99.999%, Air Liquide Gas AB, Malmö, Sweden).

**Figure 1:**
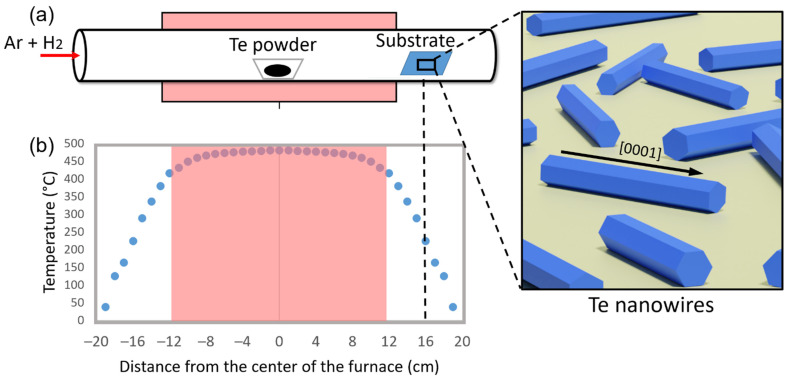
(**a**) Experimental setup. Optimal Te nanostructure growth was found at 16 cm from the centre. (**b**) Temperature profile when 500 °C was used during the synthesis.

*Materials growth.* Te nanostructures were produced via PVD. SiO_2_ substrates (16 × 18 mm^2^) consisted of undoped <100> Si coated with 300 nm Wet SiO_2_ (Siegert Wafer GmbH, Aachen, Germany). The SiO_2_ substrates were pre-cleaned in acetone, isopropanol, and deionised water for 10 min in each solvent, followed by drying at 100 °C for 10 min. Afterwards, the SiO_2_ substrates were subjected to an oxygen plasma treatment (Plasma ETCH PE-100, Carson City, NV, USA) using a 250 W under O_2_ gas flow (100 sccm) for 3 min. The prepared SiO_2_ substrates were placed on top of an alumina porcelain boat located at the end of the heating zone region inside a quartz tube as shown in the schematic in Figure 1a. The furnace used for the growth was a horizontal split-type tube furnace (Nabertherm, RSH 50/250/13, Lilienthal, Germany) with a single heating zone capable of reaching temperatures of 1300 °C. The temperature profile shown in Figure 1b was evaluated with the quartz tube inside the oven and introducing an external thermocouple type-K into the oven. An amount of 0.15 g of Te powder was placed on a quartz boat inside the quartz tube in the centre of the heating zone. The system was purged with 300 sccm of Ar for 10 min. Afterwards, the system was heated at 100 °C for 10 min under 300 sccm of Ar. The system was heated up to the desired temperature in constant gas flow environment. The heating rate was carried out in two steps, the first heating rate for all experiments was 30 °C·min^−1^ until a temperature equal to 100 °C lower than the target temperature was achieved (450 °C and 500 °C). Then a second heating rate was set at 3, 6, or 30 °C·min^−1^ depending on the sample. The synthesis time was varied between 1 and 3 h in a constant Arcal 15 environment. Finally, the samples were removed after cooling the system to 100 °C. For experiments involving Bi as an additive, 0.03 g of Bi powder was placed on an alumina porcelain boat located at three different positions in the furnace (12.0, 14.5, and 16.5 cm upstream from the centre of the furnace) corresponding to temperatures 160, 270, and 380 °C. The rest of the procedure was similar to the Bi-free experiment.

*Material characterization:* The morphology was investigated using a scanning electron microscope (Carl Zeiss Merlin FESEM, AG, Germany). TEM images were taken using a FEI Titan Krios operating at 300 kV used in the cryo-mode setup and the Talos L 120 °C operating at 120 kV (both equipment from ThermoFisher Scientific, Stockholm, Sweden). High resolution transmission electron microscope (HRTEM) images and selected area electron diffraction (SAED) patterns were obtained on a Themis Z at an accelerating voltage of 300 kV (ThermoFisher Scientific, Stockholm, Sweden) using a Gatan OneView camera (AMETEK GB Limited, Leicester, UK). X-ray photoelectron spectroscopy (XPS) was carried out on an AXIS Ultra DLD electron spectrometer (KRATOS Analytical Ltd., Manchester, UK) with a monochromatic source Al Kα-line (1486.6 eV).

## 3. Results and Discussion

### 3.1. Effect of the Carrier Gas Flow Rate and System Temperature

Tellurium nanostructures were grown onto SiO_2_ substrates using PVD by placing metallic Te powder at the centre of the heating zone, as depicted in Figure 1a. We observed that a consistent growth of nanostructured Te was only achieved when placing the SiO_2_ substrates at the end of the heating zone (16 cm away from the centre), where a sharp temperature decrease is observed (more noticeable in Figure S1). This region exhibits temperatures of 246.5 ± 18.4 °C (Figure 1a and Figure S1) when the system temperature is 500 °C. We found that this configuration enhances the supersaturation of Te near the SiO_2_ substrate, forming a variety of Te nanostructures. The growth of Te nanowires was then investigated in detail by varying the system temperature, growth time, heating rate, and gas flow. A summary of the synthesis conditions are listed in Table 1.

**Figure 2:**
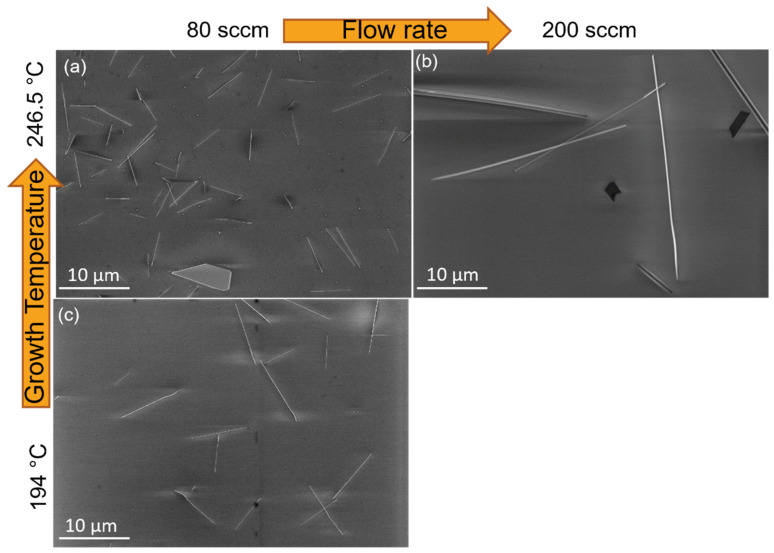
Scanning electron microscopy images of Te nanostructures growth at: (**a**) 80 sccm and 246.5 ± 18.4 °C, Te-A2, (**b**) 200 sccm and 246.5 ± 18.4 °C, Te-A3, and (**c**) 80 sccm and 194 ± 15.4 °C, Te-A4.

**Figure 3:**
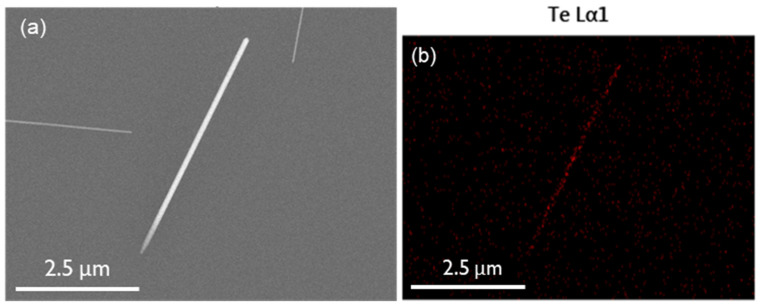
(**a**) SEM image and (**b**) EDS mapping of tellurium nanowires in sample Te-A2.

We first investigated the effect of the gas flow rate. We increased the flow rate from 50 to 200 sccm of Ar while maintaining other synthesis conditions unchanged (1 h growth time, heating rate 30/30 °C·min^−1^, furnace temperature 500 °C). The samples are labelled Te-A1, Te-A2, and Te-A3, for 50, 80, and 200 sccm of Ar, respectively. The results are summarised in Figure 2a,b. The as-produced Te nanowires were found dispersed on the SiO_2_ substrate with no preferential orientation consisting only of single tellurium metal (Figure 3). Their physical characteristics and spatial density varied significantly under the synthesis conditions. At a flow rate of 50 sccm (Te-A1), no nanostructures were found. While increasing the flow rate to 80 sccm of Ar, the Te-A2 sample exhibited several large pieces of material along the SiO2 substrate (Figure S2) with some occasional nanowires. The few produced nanowires had a diameter of 77.3 ± 28.4 nm and were 5.0 ± 1.7 μm in length, resulting in an aspect ratio (ρ = length/width) of 65. At the higher flow rate of 200 sccm, the Te-A3 sample mostly comprised nanowires with 200.8 ± 30.2 nm in diameter and 22.4 ± 9.4 μm in length (ρ = 112). As expected, higher gas carrier flow rates favoured a consistent growth of nanowires that were thicker and much larger than those seen at lower flow rates. The dimensions of the obtained nanostructures were similar to the Te nanowires reported in references [20,24]. Figure S3 shows the Te nanowires. For many wires, a nanoparticle is seen to be attached to one of their ends, indicating growth via the vapour–solid mechanism where a single Te particle serves as the seed. In general, the nucleation mechanism is based on the tendency to minimise the nanostructure total free energy [25,26], which is dependent on the surface and the interfaces, dictating orientation, radius, and length of the nanowires [20,26]. According to the vapour–solid growth mechanism, it follows that Te nanowire growth proceeds below the melting point of Te (~450 °C), which in our case, according to the temperature profile in Figure 1b, occurs at temperatures as low as 194.0 ± 15.4 °C. The effect of the gas carrier flow rate can then be understood by considering that moderate flow rates of 200 sccm led to an optimal concentration of Te near the seeds. At 80 sccm, on the other hand, an excessive Te concentration profile is formed around the seeds, causing overgrowth, and thus just a few nanostructures are produced [21,27]. Then, at 50 sccm, only large microstructures are formed, as seen in Figure S4.

We then investigated the effect of decreasing the source temperature to 450 °C (growth temperature of 194 ± 15.4) while maintaining all other parameters unchanged. We used a flow rate of 80 sccm, and the sample was labelled Te-A4, seen in Figure 2c. By reducing the temperature by just 50 °C, the average diameter and length of the Te nanowires decreased from 200.8 nm and 22.4 μm (Te-A3) to just 53.4 ± 14.1 nm and 6.4 ± 1.4 μm, respectively. However, the aspect ratio increased from 112 to 120. Apparently, the lower temperature reduced the growth rate along both radial and longitudinal directions equally, evidenced by the ~1/4 reduction in both the diameter and length. It is important to note that, under this condition, we did not observe the formation of large Te microstructures. Interestingly, using 450 °C (sample Te-A5) as the source temperature in combination with a lower flow rate (50 sccm) resulted in no nanorods and only nanoparticles were obtained, as seen in Figure S5.

### 3.2. Effect of Growth Time and Heating Rate

**Figure 4:**
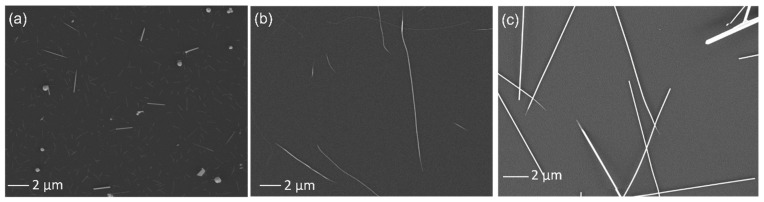
SEM images of (**a**) Te-C1 (2 h, 6 °C·min^−1^), (**b**) Te-C2 (3 h, 6 °C·min^−1^) and (**c**) Te-C3 (3 h, 3 °C·min^−1^).

The effect of growth time was investigated by increasing the synthesis time from 1 h to 2 and 3 h. For these samples, a flow rate of 80 sccm and a source temperature of 450 °C were utilised. On this occasion, we also reduced the heating rate to 6 °C·min^−1^. These samples are labelled Te-C1 and Te-C2, for 2 h and 3 h of growth time, respectively (see Figure 4a,b). After 2 h of synthesis and using a much slower heating rate of 6 °C·min^−1^ (previously 30 °C·min^−1^), the average diameter and length were significantly reduced from ~53 nm and ~6.4 μm (Te-A4) to 18.3 ± 3.7 nm and 0.91 ± 0.4 μm (ρ = 50), respectively. Meanwhile increasing the synthesis time to 3 h (Te-C2) led to nanostructures with 26.0 ± 15.9 nm diameter and 8.5 ± 2.5 μm length, giving a much larger aspect ratio ρ of 327. As expected, longer synthesis time resulted in larger nanowires if precursor feeding remained without altering the supersaturation conditions [24,28]. It is also clear that a smaller heating rate favoured the growth along the longitudinal direction, forming more wire-like structures. This parameter was seen to affect the nanorod morphology and density more drastically, therefore an additional sample with a smaller heating rate of just 3 °C·min^−1^ was prepared. The sample was labelled as Te-C3, and a SEM image can be seen in Figure 4c. We visually observed a lower yield of nanowires with fewer microstructures when compared to the Te-C2 sample (heating rate of 6 °C·min^−1^). The Te-C3 sample contained nanowires with a diameter of 54.6 ± 18.5 nm, which is twice that of the Te-C2 sample, however the length (8.6 ± 0.9 μm) remained practically unchanged, resulting in a smaller ρ of 157. A summary of the length and diameter is listed in Table 1.

**Figure 5:**
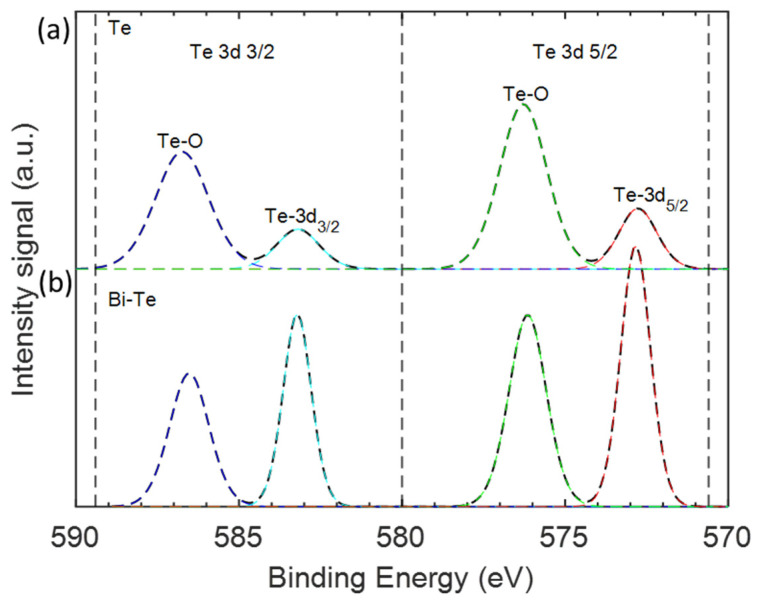
High-resolution XPS core-level spectra of (**a**) Te-C3, and (**b**) Bi-assisted Te-C3 (Bi-Te-C3). The coloured dashed lines indicate their individual contribution after deconvolution of the XPS spectra.

The most common parameter to control the morphology of nanowires is the degree of supersaturation [27]. In our work, this is controlled by the temperature gradient in the experimental setup (Figure 1), the gas flow rate, system temperature, and heating rate. We have found the optimum parameters to be a flow rate of 80 sccm, 450 °C temperature of the system with a heating rate of 3 °C·min^−1^, and 3 h of growth time. These were selected due to a higher yield of nanowires with fewer microstructures while still having a large aspect ratio. We therefore concluded that the sample Te-C3 had the optimum nanowires and proceeded to evaluate their chemical states and composition by XPS studies (Figure 5a). XPS is a surface sensitive technique that allows analysis of the photo-emitted electrons from the core level and valence band of diverse materials. The XPS spectra contain unique features that can identify the elements that occupy the surface, as well as their chemical states. In Figure 5a, two peaks are observed at 583.2 eV and 572.7 eV originating from Te-3d3/2 and Te-3d5/2, respectively. Two other features seen at higher binding energies (576.2 eV and 586.7 eV) are attributed to the oxide phase of Te [29,30]. The ratio between the oxidised peaks and Te-3d peaks reveals a high proportion of Te oxides (Te-O/Te-3d3/2 = 2.94 and Te-O/Te-3d5/2 = 2.73) since Te tends to oxidize fast when exposed to the ambient atmosphere [31,32].

**Figure 6:**
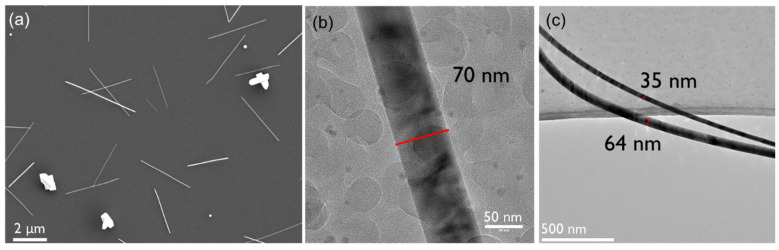
SEM (**a**) and TEM (**b**,**c**) images of Bi-Te-C3. The red lines in (**b**,**c**) indicate the regions where the diameter of the nanowires was measured.

We found a simple way to reduce the oxidation of the as-produced nanowires by introducing bismuth during the synthesis process. For this, Bi powder was placed 14.5 cm away from the centre of the oven, just before the heating zone starts. Using the same synthesis conditions as sample Te-C3, the bismuth assisted analogue Bi-Te-C3 contained nanowires with similar diameters (49.1 ± 16.2 nm), but with half the length (3.4 ± 0.7 µm) of that seen in Te-C3. Besides this, the morphology was not altered significantly, as seen in Figure 6. More importantly, a significant difference in the chemical oxidation states of Te is observed in Figure 5b, manifested by a reduction of the Te-O/Te-3d_3/2_ ratio from 2.94 to 0.69, and the Te-O/Te-3d_5/2_ ratio from 2.73 to 0.73. This indicates that Bi mitigates the formation of tellurium oxides in the nanowires. From the XPS data, we cannot directly observe the Bi atoms, indicating that the doping level lies below the detection limit of the XPS, which is roughly 0.5–1.0%. Using different evaporation temperatures for the Bi powder did not result in a change in the doping level, indicating that the doping likely only occurs at the surface of the Te wires. It is likely, however, that Bi, even at low doping levels, withdraws electrons from the Te atoms and thereby makes them less prone to oxidation.

**Figure 7:**
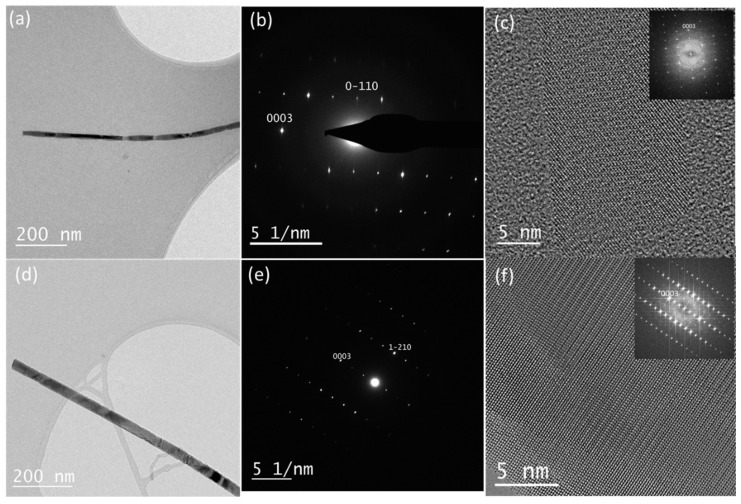
TEM, SAED, and HRTEM images of (**a**–**c**) Te-C3, and (**d**–**f**) Bi-Te-C3. The numbers in the SAED and FFT images indicate crystal directions of Te.

A detailed structural characterization of the growth direction of Te nanowires was performed using HRTEM and SAED. Both samples Te-C3 and Bi-Te-C3 were investigated, and the results are shown in Figure 7. Electron diffraction studies (Figure 7b,e) revealed the single crystalline nature of the nanowire, seen in Figure 7a,d. The SAED pattern in Figure 7b can be indexed as (100) zone axis of trigonal Te and Figure 7e as (210) zone axis, and the growth direction of the nanowire is along the (0001) in both cases. From the SAED patterns, we can conclude that there is no significant variation in the *d*-spacing along the [0001] direction for both Te-C3 and Bi-Te-C3 samples, where both samples exhibit a similar *d*-spacing of 0.59 nm. For comparison, the lattice parameter along the [0001] direction of trigonal Te is 0.596 nm [27,33]. The HRTEM images in Figure 7c,f also show the highly crystalline nature and the FFT (Fast Fourier Transform) agrees with the SAED studies. From these results, we conclude that both Te-C3 and Bi-Te-C3 samples are highly crystalline nanowires.

## 4. Conclusions

One-dimensional high-quality tellurium nanowires were synthesised via physical vapour deposition. By tuning the synthesis time, temperature, heating time, and gas flow rate, it is possible to control the diameter and length of the nanostructures. Te nanowires grow along the [0001] direction via the vapour–solid mechanism, where the supersaturation can be tuned to favour radial or longitudinal growth, effectively affecting the aspect ratio. Under optimal conditions, an aspect ratio of 327 was obtained for nanowires measuring 26 nm in diameter and 8.5 μm in length. The use of bismuth reduced the presence of tellurium oxides by half without significantly affecting their morphology.

## Figures and Tables

**Table 1:** Experimental parameters used during synthesis and obtained diameter, length, and aspect ratio.

Sample	System Temperature (°C)	Growth Temperature (°C)	Time (h)	Gas Flow (sccm)	Initial/Final Heating Rate (°C/min)	Diameter (nm)	Length (μm)	Aspect Ratio
Te-A1	500	246.5 ± 18.4	1	50	30/30	-	-	-
Te-A2	500	246.5 ± 18.4	1	80	30/30	77.3 ± 28.4	5.0 ± 1.7	65
Te-A3	500	246.5 ± 18.4	1	200	30/30	200.8 ± 30.2	22.4 ± 9.4	112
Te-A4	450	194.0 ± 15.4	1	80	30/30	53.4 ± 14.1	6.4 ± 1.4	120
Te-A5	450	194.0 ± 15.4	1	50	30/30	-	-	-
Te-C1	450	194.0 ± 15.4	2	80	30/6	18.3 ± 3.7	0.9 ± 0.4	49
Te-C2	450	194.0 ± 15.4	3	80	30/6	26.0 ± 15.9	8.5 ± 2.5	327
Te-C3	450	194.0 ± 15.4	3	80	30/3	54.6 ± 18.5	8.6 ± 0.9	157
Bi-Te-C3	450	194.0 ± 15.4	3	80	30/3	49.1 ± 16.2	3.4 ± 0.7	69

## Data Availability

Data is contained within the article or supplementary material.

## Supplementary Materials

The following supporting information can be downloaded at: https://www.mdpi.com/article/10.3390/nano12234137/s1. Figure S1: Temperature profiles, Figures S2–S5: SEM images.
