# Supplementary material for: Controlled Synthesis of Tellurium Nanowires
Source: Nanomaterials (Basel). 2022 Nov 23;12(23):4137. doi: 10.3390/nano12234137 (PMC9741254; doi:10.3390/nano12234137)
Supplement: Supplementary file 1 [file nanomaterials-12-04137-s001.zip › nanomaterials-2022662-supplementary.pdf]

Supplementary Information

Controlled Synthesis of Tellurium Nanowires

Vladimir Miranda La Hera [1], Xiuyu Wu [1], Josué Mena [1], Hamid Reza Barzegar [1], Anumol Ashok [2], Sergey Koroidov [3], Thomas Wågberg [1,*] and Eduardo Gracia-Espino [1,*]

[1] Department of Physics, Umeå University, 901 87 Umeå, Sweden
[2] Department of Materials and Environmental Chemistry, Stockholm University, 106 91 Stockholm, Sweden
[3] Department of Physics, Stockholm University, 106 91 Stockholm, Sweden
[*] Correspondence: thomas.wagberg@umu.se (T.W.); eduardo.gracia@umu.se (E.G.-E.)

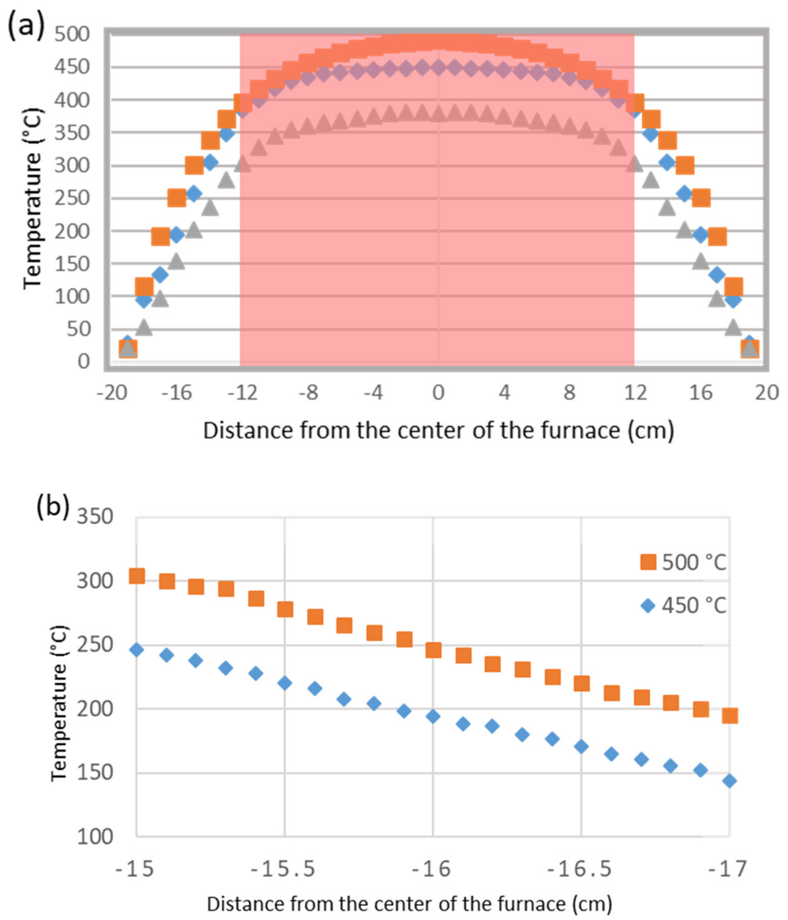

Figure S1. (a)Temperature profile in the experimental setup for different temperatures 500 °C (orange squares), 450 °C (blue diamonds), and 380 °C (gray triangles). (b) Detail temperature profile near 16cm. The red square in (a) represent the hot zone of the oven.

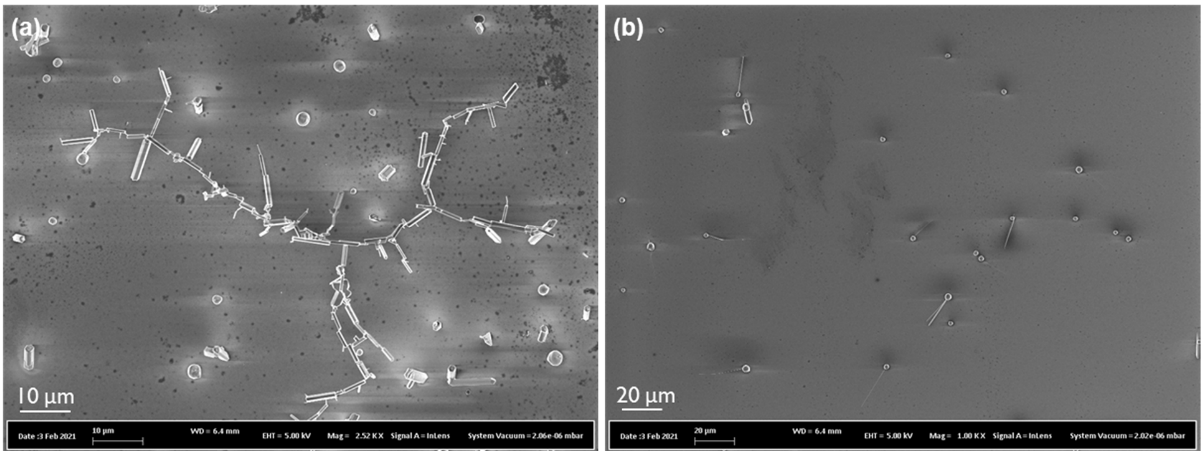

Figure S2. SEM images of sample Te-A2 taken with (a) 2.5KX and (b) 1.0KX magnifications.

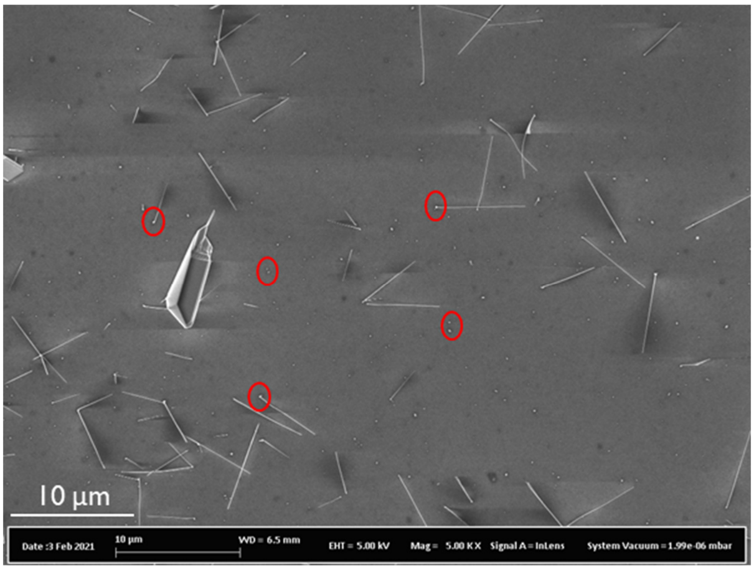

Figure S3. SEM image on sample Te-A2. Red circles denote the nanoparticles giving rise to the nanowires.

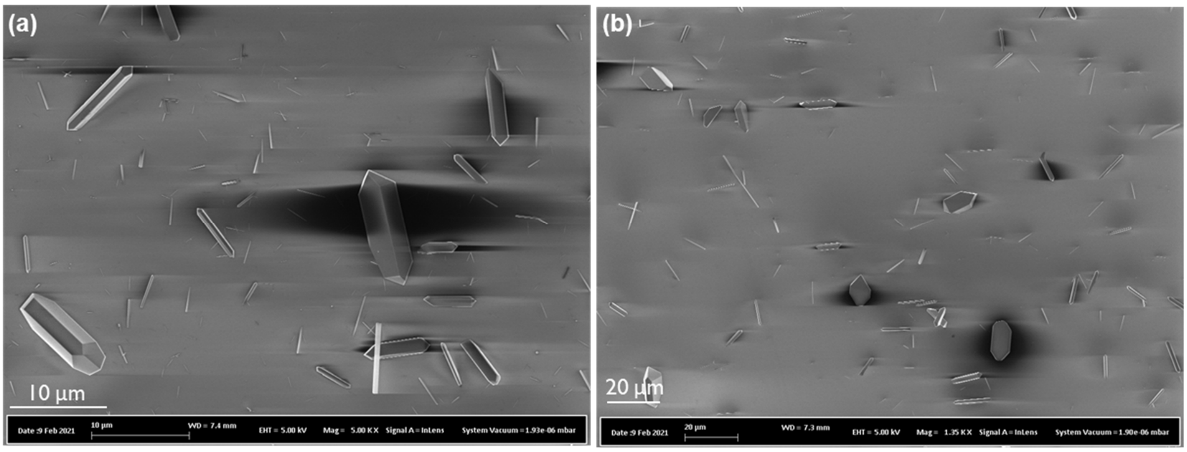

Figure S4. SEM images of sample Te-A1.

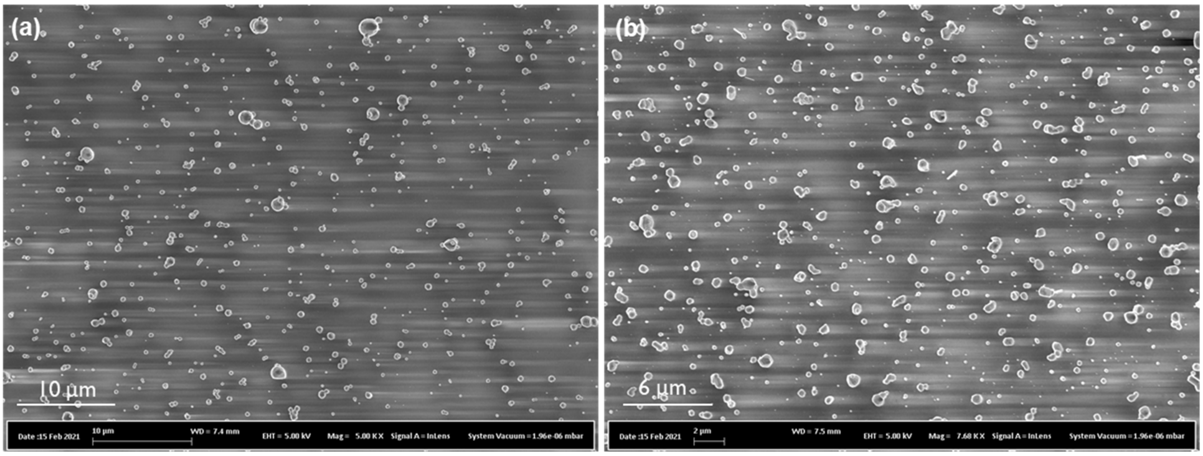

Figure S5. SEM images of sample Te-A5
